# Forensische Radiologie: Einführung und Übersicht

**DOI:** 10.1007/s00117-024-01365-2

**Published:** 2024-09-06

**Authors:** Silke Grabherr, Jochen Grimm

**Affiliations:** 1 Institut für Rechtsmedizin Lausanne-Genf, Universitätsspital Lausanne, Universität Lausanne, Universitätsspital Genf, Universität Genf, Chemin de la Vulliette 4, 1000 Lausanne, Schweiz; 2 Universitätsinstitut für Neuroradiologie, Christian-Doppler-Klinik, Paracelsus Medizinische Privatuniversität, Salzburg, Österreich

**Keywords:** Forensische Bildgebung, Postmortale Computertomographie, Postmortale Magnetresonanztomographie, Minimal-invasive Autopsie, Klinische Rechtsmedizin, Forensic imaging, Post-mortem computed tomography, Post-mortem magnetic resonance tomography, Minimally invasive autopsy, Clinical forensic medicine

## Abstract

**Hintergrund:**

In der Rechtsmedizin ist die Dokumentation von Befunden essenziell. Während einer Obduktion erfolgt dies zumeist durch die Fotografie. Jedoch gibt es zahlreiche Verletzungen, die auch bei einer klassischen Obduktion unentdeckt bleiben. In den letzten Jahren wuchs in zahlreichen Ländern zur Verbesserung der Befunddokumentation und zur Steigerung der Qualität der postmortalen Untersuchung der Stellenwert der forensischen Radiologie.

**Methoden:**

Während viele Methoden, wie die konventionelle Röntgenbildgebung oder die Computertomographie (CT) relativ einfach in den postmortalen Bereich übernommen werden können, gibt es andere Methoden, die sich schwieriger anpassen lassen. So benötigt z. B. die Durchführung einer postmortalen Angiographie ein spezifisches Konzept, welches das Auffüllen des Blutgefäßsystems und das Zirkulieren eines Kontrastmittels erlaubt. Auch die Durchführung einer postmortalen Magnetresonanztomographie (MRT) ist eine Herausforderung, da der Bildkontrast stark von der Temperatur des zu untersuchenden Körpers abhängt. Beim Einsatz an lebenden Personen, im Bereich der „klinischen Rechtsmedizin“, gibt es weitere Elemente zu beachten. So stellt sich insbesondere die Frage, welche radiologischen Methoden ohne klinische Indikation für rein rechtsmedizinische Fragestellungen zulässig sind.

**Schlussfolgerung:**

Dieser Übersichtsartikel soll die verschiedenen Methoden der forensischen Radiologie, ihre Einsatzbereiche sowie ihre Vor- und Nachteile erläutern. Außerdem werden wichtige historische Entwicklungen zum Einsatz der forensischen Radiologie und zu ihrer aktuellen Verbreitung im deutschsprachigen Raum sowie zu aktuellen und zukünftigen Entwicklungen beschrieben. Dank dieser Informationen und einer zusammenfassenden Übersichtstabelle können klare Hinweise und Empfehlungen zum Einsatz der forensischen Radiologie in der Praxis erhalten werden.

## Abstract (en)

**Background:**

In forensic medicine, documentation of findings is essential. During an autopsy, this is usually achieved by photography. However, there are numerous injuries that remain undetected even during a classic autopsy. In recent years, the importance of forensic radiology has grown in many countries to improve the documentation of findings and to increase the quality of post-mortem examinations.

**Methods:**

While many methods, such as conventional X‑rays or computed tomography, can be transferred quite easily to the post-mortem field, there are other methods that are more difficult to adapt. For example, performing a post-mortem angiography requires a specific concept that allows the vascular system to be filled and a contrast agent to circulate. Performing post-mortem magnetic resonance imaging is also a challenge, as image contrast depends on the temperature of the body being examined. When applying forensic radiology on living persons in the field of “clinical forensic medicine”, there are further elements to consider. In particular, the question arises if radiological methods are acceptable for purely forensic medical purposes without a clinical indication.

**Conclusion:**

This overview article is intended to explain the various methods of forensic radiology, their areas of application, and their advantages and disadvantages. It also describes important historical developments in the use of forensic radiology and its current spread in German-speaking countries as well as current and future developments. Thanks to this information and a summarizing overview table, clear indications and recommendations for the use of forensic radiology in practice can be obtained.

Seit Anbeginn der Rechtsprechung war es klar, dass es für gewisse juristische Fragestellungen und die daraus resultierenden Entscheidungen die Hilfe von Medizinern braucht. Schon lange vor unserer Zeitrechnung wurden Mediziner von Gerichten und Richtern hinzugezogen, um Fragen bezüglich einer Todesursache oder Verletzungen zu beantworten, für welche eine Fremdeinwirkung verantwortlich sein könnte.

Papyrusrollen aus der Quin-Dynasty (221 bis 206 v. Chr.) bezeugen, dass es schon zu diesem Zeitpunkt aktive rechtsmedizinische Tätigkeiten in Asien gab [1]. Eine wahre Revolution war das Buch „Washing away of wrongs“ von Sung Tz’u aus dem 12. bis 13. Jhd., welches in zahlreiche Sprachen übersetzt wurde und schon damals das Vorgehen bei rechtsmedizinischen Untersuchungen, inklusive von Obduktionen und der Beschreibung der dabei entstandenen Befunde dokumentierte. In Europa wird häufig die Herausgabe des Bandes „Quaestionum Medico-Leaglium“ von Pauli Zacchiae als Grundstein der Rechtsmedizin beschrieben. Dieses erschien jedoch erst im 16 Jhd. [1].

Seither hat sich die Rechtsmedizin oder „Gerichtsmedizin“, wie sie noch heute in Österreich heißt, stark weiterentwickelt. Die deutschsprachige Schule der Rechtsmedizin war in ganz Europa bekannt. Unter den berühmtesten Vertretern fanden sich Carl von Rokitansky (1804–1878), Eduard Ritter von Hofmann (1837–1897) sowie Rudolph Ludwig Carl Virchow (1821–1902). Ihre Vertreter hatten regen Austausch und kamen aus Deutschland sowie dem ehemaligen Österreich-Ungarischem Reich. Im Jahre 1904 wurde schließlich in Breslau auf Vorschlag von Fritz Strassmann hin die „Deutsche Gesellschaft für Gerichtsmedizin“ (heute: „Deutsche Gesellschaft für Rechtsmedizin“) gegründet [1].

Forschung, Weiterbildung und Austausch waren immer schon Leitmotive der Gesellschaft. Wie auch andere Gebiete der Medizin entwickelt sich die Rechtsmedizin laufend weiter. Somit ist es nicht verwunderlich, dass die Rechtsmedizin auch stark von der klinischen Medizin beeinflusst wird, welche seit der Entdeckung der Röntgenstrahlen auf bildgebende Methoden zurückgreifen kann. Schon ganz zu Beginn des Einsatzes der Röntgenstrahlen wurden diese für forensische, also gerichtliche Fragestellungen benutzt [2], so z. B. zum Auffinden von Projektilen nach Schussverletzungen bei lebenden, aber auch verstorbenen Personen. Die forensische Radiologie ist deshalb schon beinahe genauso alt wie die Radiologie selbst.

Die *moderne forensische Radiologie* ist allerdings um einiges jünger. Heutzutage zählt hierzu der Einsatz verschiedener radiologischer Methoden, für forensische, d. h. rechtsmedizinische oder spurenkundliche Zwecke (Tab. 1). Zu diesen Methoden zählen der Einsatz von konventioneller Röntgenbildgebung (KR), Computertomographie (CT), Ultraschall (US) und Magnetresonanztomographie (MRT). Diese Modalitäten können mit minimal-invasiven Methoden kombiniert werden. Dabei sind die Durchführung einer postmortalen Angiographie (PMA) oder von bildgesteuerten Probenentnahmen zu histologischen, toxikologischen, molekularbiologischen oder anderen Untersuchungen zu nennen. Letztere werden häufig unter dem Begriff der minimal-invasiven Autopsie (MIA) zusammengefasst.

**Tab. 1:** Übersichtstabelle der verschiedenen Methoden der radiologischen Bildgebung mit deren Haupteinsatzgebieten in der postmortalen und der klinischen Rechtsmedizin sowie den entsprechenden Literaturhinweisen

Methode	Einsatz	Literatur
Konventionelle Röntgenbildgebung (KR)	*Untersuchung von Lebenden:*	[9–11]
	Suche nach Fremdkörpern (Projektilen, Drogen etc.)	
	Forensische Altersdiagnostik	
	*Postmortale Untersuchungen:*	
	Körper, die nicht in eine CT passen (zu schwer, zu groß, im Sarg etc.)	
Computertomographie (CT)	*Untersuchung von Lebenden:*	[8, 11–19]
	Suche nach Fremdkörpern (Projektilen, Drogen etc.)	
	Forensische Altersdiagnostik	
	*Postmortale Untersuchungen:*	
	Screeningmethode zur Dokumentation des Leichnams, Vorbereitung der Obduktion und Stellen der Indikation für weitere (bildgebende) Untersuchungen	
	Suche nach Fremdkörpern (Projektilen, Implantaten etc.), insbesondere bei Fäulnisleichen und zur Identifizierung des Leichnams	
	Darstellung von Frakturen (häufig 3D-Darstellung im Bildkatalog)	
	Darstellung von Luft (Luftembolie etc.)	
	Bildgesteuerte Probenentnahme (MIA)	
Magnetresonanztomographie (MRT)	*Untersuchung von Lebenden:*	[20–26]
	Opfer von Strangulation/Würgen zum Ausschluss innerer Verletzungen	
	*Postmortale Untersuchungen:*	
	Untersuchung des Herzens bei plötzlichem Herztod	
	Untersuchung des Gehirns bei gewaltsamem Tod (z. B. Strangulation, Erhängen, traumatische Verletzungen etc.)	
	Untersuchung von Feten und Neugeborenen oder Kleinkindern	
Ultraschall (US)	*Untersuchung von Lebenden:*	[27–29]
	Forensische Altersdiagnostik	
	*Postmortale Untersuchungen:*	
	Untersuchung der Bauchorgane	
	Bildgesteuerte Probenentnahme (MIA)	
Postmortale Angiographie (PMA)	*Hauptsächlich im Zusammenhang mit einer CT zur Untersuchung von:*	[15, 30–32]
	Plötzlichem Tod (Herztod, Aortendissektion, Aneurysma)	
	Scharfer Gewalt (Schnitt-Stichverletzungen mit Darstellung des Stichkanals)	
	Schussverletzungen (Darstellung von Schussverlauf, Projektilen etc.)	
	Tod nach medizinischem Eingriff (Darstellung einer Blutungsquelle)	
Minimal-invasive Autopsie (MIA)	Vorwiegend bei natürlichem Tod, wenn keine CT vorhanden ist, hauptsächlich Probenentnahme zur mikrobiologischen und histologischen Untersuchung (Krebsdiagnostik)	[33–35]

Werden weitere bildgebenden Methoden hinzugezogen, so wird von *forensischer Bildgebung* gesprochen. Hierzu gehören neben den Methoden der forensischen Radiologie die Fotografie und die Fotogrammmetrie [3], das 3D-Oberflächenscanning [4] und weitere bildgebende Methoden, wie z. B. die Untersuchung der Körperoberfläche mittels infraroten oder ultravioletten Lichts [5].

*Im deutschsprachigen Raum* spielt die forensische Bildgebung eine wichtige Rolle. Federführend auf diesem Gebiet ist die Schweiz, in der alle universitären Institute (Bern, Basel, Zürich, Lausanne-Genf) mit eigenem Equipment für die forensische Bildgebung ausgestattet sind. Dies besteht zumindest aus einem Computertomografen, teilweise aber auch aus einem MRT-Scanner, verschiedenen Oberflächenscannern und konventionellen Röntgengeräten. Die Durchführung einer postmortalen CT (PMCT) ist hier Standard und komplettiert die konventionelle Obduktion. Auch die postmortale Angiographie findet in bestimmten Instituten regelmäßigen Einsatz (z. B. in etwa einem Viertel der Obduktionsfälle in Lausanne-Genf).

Aber auch in Deutschland und in Österreich gewinnt die forensische Bildgebung im Allgemeinen, insbesondere die forensische Radiologie, immer mehr an Popularität. Auch hier werden zunehmend rechtsmedizinische Institute mit CT-Geräten ausgestattet. Die Deutsche Gesellschaft für Rechtsmedizin (DGRM) hat dem Beispiel der Schweizer Gesellschaft für Rechtsmedizin folgend 2014 die „Arbeitsgemeinschaft für Forensische Bildgebung“ (AGFB) gegründet [6]. Diese Arbeitsgemeinschaft hat inzwischen mehrere Projekte abgeschlossen, darunter einen Übersichtsartikel zu den Methoden der forensischen Bildgebung [7] und einen Indikationskatalog zum Einsatz der PMCT [8]. Seit der Gründung der Arbeitsgemeinschaft für Forensisch-Radiologische Bildgebung (AG FRB) der Deutschen Röntgengesellschaft (DRG) im Jahr 2020 arbeiten die beiden Arbeitsgemeinschaften Hand in Hand, um den Einsatz moderner forensischer Radiologie zu fördern, Standards zu entwickeln und Radiologen sowie Rechtsmediziner mit der komplexen Interpretation forensisch radiologischer Untersuchungen vertraut zu machen. Da zur Interpretation der forensisch-radiologischen Bilddaten nicht nur radiologisches, sondern auch rechtsmedizinisches Wissen und Erfahrung benötigt werden – jedenfalls bis zur Etablierung einer entsprechenden Subspezialisierung –, empfiehlt sich eine enge interdisziplinäre Abstimmung zwischen Radiologen und Rechtsmedizinern bei der Befundung.

## Methoden der forensischen Radiologie

Wie bereits angegeben, werden verschiedene radiologische Methoden für die forensische Bildgebung eingesetzt. Dieser Teil soll einen kurzen Einblick in die verschiedenen Einsatzgebiete geben. Eine Übersicht findet sich auch in Tab. 1.

### Konventionelle Röntgenbildgebung

Trotz des relativ hohen Alters der Methode hat die KR noch immer eine Bedeutung für die forensische Radiologie. Die Vorteile sind zum einen relativ geringe Kosten und breite Verfügbarkeit und zum anderen die hohe Ortsauflösung und (beim Einsatz am Lebenden) die geringe Strahlenbelastung. Beim Lebenden ist die KR eine wesentliche Grundlage der radiologischen Altersdiagnostik [9]. Auch zur Lokalisation und Charakterisierung von röntgendichten Fremdkörpern (Metallsplittern, Projektilen, Glas, Implantate, Drogenpäckchen etc.) ist sie gut geeignet [10], ante wie auch post mortem. Es ist allerdings zu beachten, dass es sich bei den resultierenden Bildern um eine Projektion auf eine Ebene handelt, so dass es oft schwierig ist, oder einen erhöhten Aufwand erfordert, Befunde genau zu lokalisieren. Auch kann es durch Überlagerungen zur Verdeckung relevanter Befunde kommen, was die Sensitivität der Untersuchung verringert (z. B. Übersehen von Drogenpäckchen im Darm durch Gasansammlungen; [11]).

Post mortem wird die KR zur Lokalisation und Charakterisierung röntgendichter Fremdkörper, zur Identifikation, teilweise auch zur Beurteilung knöcherner Läsionen verwendet, insbesondere dann, wenn eine CT nicht verfügbar oder aus anderen Gründen (wie z. B. Größe oder Haltung des Leichnams unter der Leichenstarre) nicht durchführbar ist.

### Computertomographie

Die CT hat einen zentralen Stellenwert in der forensischen Bildgebung. Sie ist breit verfügbar, verursacht moderate Kosten und liefert umfassende Informationen. Im Gegensatz zu den meisten anderen Methoden ermöglicht sie die zusammenhängende Darstellung des gesamten Körpers in kurzer Zeit. Sie ist hervorragend geeignet, um Fremdkörper zu lokalisieren und zu charakterisieren, wobei die dreidimensionale Darstellung Vorteile bei der genauen Lokalisierung gegenüber der KR bietet. Beim Einsatz am Lebenden muss aus Strahlenschutzgründen der persönliche Nutzen des Einzelnen bzw. der Gesellschaft gegen die Risiken der Strahlenanwendung abgewogen werden. Beim Post-mortem-Einsatz gibt es im Gegensatz zur MRT praktisch keine Kontraindikationen gegen die CT, in Einzelfällen kann sie jedoch am Umfang oder der atypischen Haltung des Patienten bzw. Leichnams scheitern, solange die Totenstarre besteht.

Beim Lebenden wird die CT z. B. zur Detektion von Drogenpäckchen bei Drogenkurieren verwendet [11, 12]. Diese Untersuchung ist deutlich sensitiver als die KR [11]. Auch in der Altersdiagnostik am Lebenden hat die CT einen Stellenwert [9] und wird zur Bestimmung der verschiedenen Entwicklungsstadien der Schlüsselbeinepiphysen genutzt. Zudem lassen sich verschiedene Tat- oder Unfallfolgen, insbesondere Frakturen, gut charakterisieren und dokumentieren.

Post mortem liefert die CT als Grundlage der weiteren Diagnostik die dreidimensionale Darstellung des gesamten Leichnams. Hierdurch können Fremdkörper lokalisiert und das weitere diagnostische Prozedere geplant werden. Falls eine Post-mortem-MRT geplant ist, ist ggf. eine CT sinnvoll, um metallische Fremdköper auszuschließen, die eine Kontraindikation für eine MRT darstellen würden, z. B. weil sie sich aufgrund des Magnetfeldes verlagern könnten. Für eine Identifikation des Verstorbenen können ebenfalls wichtige Informationen – beispielsweise über Implantate oder die Konfiguration der Nasennebenhöhlen – gewonnen werden [13, 14]. Die PMCT liefert zudem regelmäßig wertvolle Informationen über relevante pathologische Veränderungen, die teilweise bei der Autopsie schwierig zu detektieren sind [15], insbesondere Gasansammlungen (z. B. Pneumothorax, Gasembolie) und Knochenverletzungen. Auch bei fortgeschrittener Verwesung können noch relevante Befunde erhoben werden [16].

In Einzelfällen lässt sich bereits anhand der PMCT eine Aussage über Todesart und Todesursache treffen [16, 17]. In den übrigen Fällen kann die PMCT Hinweise liefern, welche weiterführenden diagnostischen Maßnahmen sinnvoll sind (PMCT als Triage-Tool [15, 18, 19]) oder welchen Körperregionen in einer folgenden Autopsie besondere Aufmerksamkeit gewidmet werden sollte. Auch zur Planung eines minimal-invasiven Verfahrens, z. B. einer Probenentnahme, eignet sich die PMCT.

### Magnetresonanztomographie

Aufgrund der begrenzten Verfügbarkeit, der aufwändigen und langwierigen Untersuchung, der komplexen Interpretation und der hohen Kosten wird die MRT in der forensischen Radiologie insgesamt relativ selten eingesetzt, in einzelnen Zentren jedoch durchaus mit relevantem Erfolg.

Die MRT kann kontraindiziert sein, insbesondere im Fall von metallischen Implantaten oder Fragmenten, die während der Untersuchung ihre Lage verändern oder sich erwärmen können. Auch kann der häufig im Vergleich zur CT geringere Öffnungsdurchmesser des Gerätes ein Hindernis für die Untersuchung darstellen. Eine Besonderheit der Post-mortem-MRT ist die Temperaturabhängigkeit des Bildkontrastes, was sowohl die Durchführung der Untersuchung als auch die Interpretation der Bilder deutlich erschwert [20].

Gegenüber der CT liefert die MRT einen überlegenen Weichteilkontrast, sie ist ihr jedoch unterlegen bei der Darstellung von Knochen und Gas. Als radiologische Methode ohne ionisierende Strahlung hat die MRT insbesondere bei der forensischen Diagnostik am Lebenden eine besondere Bedeutung, mittels MRT werden häufig Tat- und Traumafolgen diagnostiziert und dokumentiert [21, 22]. Um eine Strahlenexposition der zu Untersuchenden zu vermeiden, wird auch aktiv an Methoden zur forensischen Altersdiagnostik mittels MRT geforscht [23, 24].

Post mortem wird die MRT insbesondere häufig bei Feten und Neugeborenen oder Kleinkindern verwendet, wobei das Untersuchungsfeld relativ klein und damit die Untersuchungszeit noch relativ kurz ist [25]. Auch bei der Beurteilung kardialer Veränderungen wird sie verwendet, beispielsweise werden ihre diagnostischen Möglichkeiten bei Verdacht auf plötzlichen Herztod intensiv erforscht [26]. Auch bei ausgeprägten Verwesungserscheinungen lassen sich mit der MRT häufig noch relevante Informationen gewinnen.

### Ultraschall

Trotz breiter Verfügbarkeit und geringer Kosten hat der US in der forensischen Bildgebung eine untergeordnete Bedeutung. Er ist gut geeignet, oberflächliche Strukturen darzustellen, auch Frakturen, Organläsionen, Flüssigkeits- und Gasansammlungen lassen sich je nach Lokalisation gut detektieren. Ähnlich wie in der klinischen Medizin lassen sich darstellbare Strukturen gut ultraschallgezielt punktieren und beproben [27, 28]. In Einzelfällen lässt sich auch mittels US die Todesursache ermitteln [28]. Wesentliche Limitationen sind die begrenzte Eindringtiefe und die Intransparenz von Knochen und Gas. Auch ist die US-Untersuchung stärker vom Untersucher abhängig als andere forensisch-radiologische Verfahren. Die Untersuchung größerer Körperabschnitte ist zudem zeitaufwändig. Da regelmäßig andere radiologische Methoden als primäre Bildgebung verwendet werden (insbesondere CT und MRT), welche die genannten Vorteile ebenfalls bieten, wird US vor allem dort eingesetzt, wo CT und MRT nicht verfügbar sind oder aus anderen Gründen (z. B. Kosten) nicht eingesetzt werden können.

Eine weitere Anwendungsmöglichkeit des US ist wiederum in der forensischen Altersschätzung am Lebenden zu sehen [29]. Wie auch die MRT, soll sie eine alternative Methode zu Techniken mittels Röntgenstrahlung darstellen, was die Forschung auf diesem Gebiet stark motiviert.

### Postmortale Angiographie

Die PMA (Visualisierung der Gefäße) kann auf eine lange Historik zurückblicken. Ursprünglich kommt sie nämlich aus dem Gebiet der Anatomie und wurde bereits im 16. Jhd. eingesetzt [30]. Zu dieser Zeit wurden sog. anatomische Gefäßausgüsse erstellt, die dazu dienen sollten, die Morphologie des menschlichen Gefäßsystems zu verstehen. Heißes Wachs oder andere Substanzen, die nach Einfüllung in die Gefäße aushärten, wurden eingesetzt, und nach Entfernung des umliegenden Gewebes konnten diese Ausgüsse studiert werden. Im 19. Jhd. fanden sich zahlreiche „Rezepte“ zur Erstellung solcher Injektionsgemische. Nach Entdeckung der Röntgenstrahlen wurden den verschiedenen Mischungen recht schnell röntgendichte Substanzen wie Bleipartikel oder Anderes hinzugefügt, so dass die Gefäße nicht nur nach Mazerieren des Gewebes, sondern bereits im Präparat, auf dem Röntgenbild sichtbar waren [30].

Mit Einführung der modernen postmortalen Bildgebung, wurde auch das Thema der PMA wieder aktuell. Verschiedene Arbeitsgruppen arbeiteten Anfang dieses Jhd. an der Entwicklung von Techniken zur PMA mittels CT. Diese neuen Methoden können in „Ganzkörper PMA-Methoden“ und in „Lokalisierte PMA-Methoden“ eingeteilt werden, wovon Letztere hauptsächlich die Darstellung der Herzkranzgefäße (Koronar-PMA) zum Ziel haben. Je nach Kontrastmittel können auch PMA mittels öliger Gemische von solchen mittels wasserlöslicher Kontrastmittel unterschieden werden. Wasserlösliche Kontrastmittel kommen hauptsächlich bei der lokalisierten PMA (Kononar-PMA) zum Einsatz. Bei der Verwendung zu einer Ganzkörper-PMA werden ihnen hygroskopische Substanzen, hauptsächlich Polyethylenglykol (PEG), beigemischt, um ein zu starkes Austreten aus dem Gefäßsystem zu vermeiden. Übersichten zu den verschiedenen Methoden, Einsatzgebieten sowie Vor- und Nachteilen der PMA finden sich zahlreich in der Literatur [31]. Wichtig ist zu unterstreichen, dass die modernen Methoden der PMA, insbesondere die am häufigsten angewandte Methode der „Multi-phase Post-Mortem Computed Tomography Angiography (MPMCTA)“ nach Grabherr et al. [32], der klassischen Obduktion bei der Darstellung von Gefäßbefunden überlegen sind. Die MPMCTA gilt deshalb als Referenzstandard zur Darstellung von Blutungsquellen und zur Erkennung einer modifizierten Gefäßanatomie (z. B. nach koronarer Bypassoperation; [15]).

### Minimal-invasive Autopsie

Als MIA kann jede postmortale Bildgebung bezeichnet werden, die auch eine minimal-invasive Komponente aufweist. Das heißt, dass die PMA eigentlich auch eine Form von minimal-invasiver Autopsie darstellt, da die Einspritzung des Kontrastmittelgemisches eine invasive Maßnahme erfordert (zumeist Kanülierung eines Blutgefäßes oder zumindest Einspritzung mittels einer Injektionsnadel). Dennoch wird dieser Begriff heutzutage zumeist zur Beschreibung von postmortaler Bildgebung und gleichzeitiger Entnahme von Proben eingesetzt.

Meistbeschrieben ist die MIA im Zusammenhang mit US-Untersuchungen [33]. Dabei wird dank bildgesteuerter Probenentnahme Material für histologische, toxikologische, klinisch-chemische, mikrobiologische oder genetische Untersuchungen gewonnen. Hauptsächlich dient sie zur Durchführung klinischer pathologischer Obduktionen, bei denen entweder die Tumordiagnostik oder die virale Diagnostik (im Rahmen der COVID-19-Pandemie) im Vordergrund steht [34].

In der Schweiz, im Rahmen des Virtopsy®-Projekts, wurde eine umfassende Form der MIA präsentiert. Dank automatisiertem Equipment, dem sog. Virtobot®, soll der Leichnam vollständig digitalisiert und untersucht werden [35]. Dieser Roboter, welcher an die PMCT gekoppelt ist, führt einen 3D-Oberflächensan mittels eines 3D-Sanners durch. Die erhaltenen Oberflächendaten können anschließend mit den PMCT-Daten fusioniert werden. Außerdem können bildgesteuert mittels des Roboters biologische Proben aus dem Leichnam entnommen werden. Auch das Kontrastmittel zur PMA soll durch den Roboter injiziert werden. Bisher fehlen jedoch die praktischen Daten, um die wahre Eignung des Roboters zur Durchführung reeller Fälle analysieren zu können.

## Diskussion

Die forensische Bildgebung als mittelbare Untersuchungsmethode hat in den meisten Fällen eine unterstützende Funktion in der Rechtsmedizin. Sie ergänzt die unmittelbaren Untersuchungsmethoden, das heißt, die klinisch-forensische Untersuchung am Lebenden sowie äußere Leichenschau und Obduktion am Verstorbenen. Ihren nicht abzustreitenden Nachteilen, insbesondere zusätzlichen Kosten, Zeitverlust, notwendiger Geräteausstattung und Personalverfügbarkeit, stehen erhebliche Vorteile gegenüber. So liefert die Bildgebung objektive Befunde, die vom Untersucher relativ unabhängig sind. Die erhobenen Bilddaten dokumentieren den Status quo im Moment der Untersuchung und können ohne zeitliche Begrenzung archiviert werden. Dies erlaubt auch nach Jahren noch eine Nach- oder Zweitbegutachtung, was bei den unmittelbaren Untersuchungsmethoden nicht gegeben ist. Auch lassen sich Befunde anhand von Bilddaten dem medizinischen Laien (z. B. den Geschworenen oder dem Richter) leichter und weniger belastend erklären, als es mit einem Obduktionspräparat oder einer Fotografie des Leichnams möglich wäre. Räumliche Zusammenhänge, z. B. ein Schusskanal, können anhand der CT des noch weitgehend unmanipulierten Körpers häufig leichter nachvollzogen werden als am geöffneten Leichnam während der Obduktion. Manche Befunde werden auch erst durch die Bildgebung erkennbar, so dass insgesamt die Qualität der forensischen Diagnostik durch die Bildgebung gesteigert wird.

Wenn aus religiösen oder ethischen Gründen eine Obduktion abgelehnt wird, oder aus anderen Gründen nicht durchgeführt werden kann, ist die forensische Bildgebung als nicht- bzw. minimal-invasive Methode häufig eine akzeptierbare Alternative. Auch wenn sie idealerweise in Kombination mit der Obduktion angewandt werden sollte und sie als alleinige Untersuchung weniger sensitiv ist, so liefert sie doch häufig wichtige Informationen zu Todesart und Todesursache.

## Fazit für die Praxis

- Die moderne forensische Radiologie umfasst die Anwendung verschiedener Methoden für rechtsmedizinische oder spurenkundliche Zwecke und bietet hier viele Vorteile.
- Neben der konventionellen Röntgenbildgebung und der Computertomographie (CT) kommen auch Ultraschall (US), Magnetresonanztomographie (MRT) sowie die Angiographie zum Einsatz.
- Alle Verfahren sind am Lebenden wie auch post mortem, wenn in manchen Fällen auch mit einigem Aufwand, möglich.
- Die postmortale Angiographie (PMA) und bildgesteuerte Probenentnahmen zu histologischen, toxikologischen, molekularbiologischen oder anderen Untersuchungen werden auch als minimal-invasive Autopsie (MIA) zusammengefasst.
- Die forensische Bildgebung hat heutzutage einen festen Stellenwert in der modernen forensischen Praxis.
- Mit zunehmender Verbreitung und Standardisierung ist damit zu rechnen, dass die Bedeutung der forensischen Bildgebung im Allgemeinen und der forensischen Radiologie im Speziellen weiter zunehmen wird.
